# Correlation between the Expression of PD-L1 and Clinicopathological Features in Patients with Thymic Epithelial Tumors

**DOI:** 10.1155/2018/5830547

**Published:** 2018-04-23

**Authors:** Yanmei Chen, Yuping Zhang, Xiaoling Chai, Jianfang Gao, Guorong Chen, Weifen Zhang, Yunxiang Zhang

**Affiliations:** ^1^Department of Pathology, Wenzhou People's Hospital, Wenzhou, Zhejiang 325000, China; ^2^Department of Pathology, Weifang People's Hospital, Weifang, Shandong 261041, China; ^3^Department of Pathology, The First Affiliated Hospital, Wenzhou Medical College, Wenzhou, Zhejiang 325000, China; ^4^School of Pharmacy, Weifang Medical University, Weifang, Shandong Province 261053, China

## Abstract

The incidence of thymic epithelial tumors (TETs) in the Chinese population was much higher than that in the North American population. In clinical treatment, the prognosis of benign tumors after surgical resection was significantly better than that of malignant tumors. Currently, the commonly used clinical indicators for TET staging included Masaoka staging and WHO (2015) pathological criteria; however, the distinction between the benign and malignant tumors and diagnosis is yet to be explored. The current study demonstrated that the expression of PD-L1 in tumor cells was correlated with the degree of TET malignancy. The quantitative analysis of PD-L1 expression in 70 cases of TET tumor samples revealed that the positive rate of PD-L1 expression in types A, AB, B1, and B2 of thymoma (40 cases) was 37.5% (15/40), which was significantly lower than that in type B3 thymoma and thymic carcinoma (76.67%, 30 cases, 23/30) as demonstrated by chi-square test (*P* < 0.05). In addition, the two methods were analyzed for the quantitative detection of PD-L1 expression. The results from the estimation of transcriptional RNA expression and quantitative protein immunohistochemistry were consistent (*r* = 0.745). Furthermore, we also analyzed PD-L1 expression level in different types of TETs from TCGA database and observed that higher PD-L1 expression was in thymic carcinoma than in thymoma. Therefore, it could be concluded that PD-L1 expression in TET cells was correlated with the degree of malignancy, whereas the estimation of PD-L1 expression was potentially applicable in the clinical staging of TETs.

## 1. Introduction

Thymic epithelial tumors (TETs) referred to tumors originating from thymic epithelial cells or those differentiating into the thymic epithelium, including thymoma and thymic carcinoma [1]. The incidence of thymoma in China was about 3.93/1000000, which was higher than that in the North American population (2.14/1000000). The current clinical diagnosis of benign or malignant thymic tumors was primarily carried out via the Masaoka staging and WHO (2015) pathological evaluation system. However, some of the tumors in an early stage, which was based on this classification system, still showed a relatively strong invasion [2], and, hence, surgical resection was the major therapy for such tumors. However, TETs cannot be radically cured by surgical resection for invasive thymoma, and these tumors often relapse after surgery with poor prognosis. Currently, immunotherapy based on the blockage of programmed death 1 (PD-1)/programmed death ligand 1 (PD-L1) is satisfactory in a variety of aggressive tumor species. Moreover, PD-L1 inhibited the activation of T-cells by binding to the receptor PD-1 on the surface of cytotoxic T-cells, whereby some tumors were evaded from the immune system-mediated killing through the PD-1/PD-L1 signaling pathway [3], which has been validated in animal model experiments [4]. Thus, the purpose of treating tumors can be achieved by blocking the PD-1/PD-L1 signaling pathway to induce apoptosis. The PD-1/PD-L1 pathways have been studied in a variety of tumors, such as melanoma, ovarian cancer, colon cancer, lung cancer, and kidney cancer. The expression of PD-1 and PD-L1 may be associated with clinicopathological features and poor prognosis of malignant tumors [5–8]. Clinically, the PD-1 monoclonal antibodies, Pembrolizumab and Nivolumab, have been slightly effective in the treatment of tumors such as non-small-cell lung cancer and malignant melanoma. Thymus was an immune organ for the differentiation, development, and maturation of T-cells. PD-L1 was expressed in the thymic epithelial cells in normal thymus [9, 10]. However, only a few studies concerning the PD-1/PD-L1 in TETs are yet available globally. Thus, we aimed to explore the relationship between the expression of PD-L1/PD-1 in TETs and the correlation with the clinicopathological features. Thus, we attempted to provide a novel insight into anti-PD-1/PD-L1 treatment.

## 2. Materials and Methods

### 2.1. Clinical Information

A total of 70 cases of pathologically diagnosed TETs by surgical resection or needle biopsy were collected from 2012 to 2017 at the Department of Pathology in Weifang People's Hospital and Wenzhou People's Hospital, China. The cohort consisted of 50 thymoma and 20 thymic carcinoma cases. According to the WHO (2015) diagnostic criteria, two pathologists reviewed the section, confirmed the diagnosis, and deduced that there were sufficient tumor cells for subsequent immunohistochemistry (IHC) and genetic testing. Also, the clinicopathological data, including age, gender, with/without myasthenia gravis, with/without preoperative and postoperative radiotherapy and chemotherapy, tumor size, pathological classification, Masaoka staging, with/without lymph node, and distant metastasis were collected.

### 2.2. Tissue Microarray Preparation, IHC Staining, and Interpretation of PD-L1 and PD-1

A 4 × 4 array of acceptor wax blocks was made using a tissue chip instrument. The diameter of each micropore was 0.9 mm, the interval about 1 mm, and the depth about 3 mm. The IHC staining was performed on a Roche BenchMark XT fully automated IHC instrument. PD-L1 (SP142, Ventana Medical System, Tucson, AZ, USA) and PD-1 (NAT, Ventana Medical System, Tucson, AZ, USA) staining was performed according to the manufacturers' instructions. Phosphate buffer saline (PBS) was used as a negative control instead of the primary antibody. The placental tissue and tonsil tissue were used as positive control for PD-L1 and PD-1, respectively. PD-L1 was primarily located in the cell membrane; the positive color was manifested as yellow to brown linear staining on the tumor cell membrane. PD-1 was localized in the cytoplasm of the interstitial lymphocytes. The interpretation of PD-L1 results was divided into two parts: the percentage of positive cells (A) and staining intensity (B). The percentage of positive cells (A): enumeration of the percentage of stained tumor cells (0–100%), percentage of positive cells = number of positive cells/total cells × 100%. The standard of staining intensity score for positive cells: 0 point, negative; 1 point, weakly positive (light yellow); 2 points, moderately positive (brownish yellow); 3 points, strongly positive (tan). The expression of PD-L1 was evaluated by the product of positive cell percentage and the staining intensity, and the positive expression was defined when the above formula-based estimation was ≥3% [11]. The interstitial lymphocyte PD-1 in the thymoma tissues was interpreted independently. A positive result was deemed as the percentage of positive cells ≥ 5%, while the negative result was defined as <5% [12].

### 2.3. Detection and Interpretation of PD-L1 mRNA in RT-PCR

The wax blocks containing corresponding tumor tissues were serially sectioned into 5–8 slices, each with a thickness of 5 *μ*m. Firstly, the total tissue RNA was extracted from the FFPE samples by RNA isolation and extraction kit (Tiangen Biochemical Beijing Co., Ltd.), followed by reverse transcription into cDNA and qPCR. The qPCR reaction system consisted of 2 *μ*L cDNA template, 1 *μ*L PD-L1 primers and probes (Life Technology Company, USA), 7 *μ*L ribozyme-free water, and 10 *μ*L Master Mix. The specific reaction program was as follows: Uracil-DNA Glycosylase incubation at 50°C for 2 min, polymerase activation at 95°C for 10 min, and PCR reaction at 95°C for 15 s and 60°C for 1 min for a total of 40 cycles. The gene mRNA expression level was detected based on the difference of Ct values (ΔCt) of the patient's target gene and the housekeeping gene as compared to the corresponding ΔCt database (the values in the database were fitted to a normal distribution curve; the expression was the relative position of the data on the curve), and the normal distribution frequency of the patient's ΔCt in the population was obtained. The relative expression level of the genes in this study was defined as follows: <0.25 was low expression; 0.25–0.75 was moderate expression; and >0.75 was high expression.

### 2.4. Detection of PD-L1 Expression Level in TETs from TCGA Database

105 TET cases were selected for analysis. All cases had detailed clinical information and RNA-seq results. According to the clinical information of TET patients, all patients were divided into two categories, including thymic carcinoma and thymoma. Then we analyzed PD-L1 expression level of patients with thymic carcinoma or thymoma, respectively, and the difference of PD-L1 expression level in different categories of TETs was calculated by test. *P* values ≤ 0.05 were considered to be statistically significant.

### 2.5. Statistical Analysis

Statistical analysis was performed using SPSS 21.0 (IBM Co., Armonk, NY, USA). The comparisons between the* PD-L1* mRNA expression and PD-L1 protein and between PD-L1 protein expression and clinicopathological features were conducted by chi-square or Fisher's exact test, as appropriate. The comparisons between the PD-L1 protein and PD-1 protein expression were conducted by chi-square or Fisher's exact test, as appropriate. The correlation between the variables was analyzed by Spearman correlation analysis. Two-sided *P* values ≤ 0.05 were considered to be statistically significant.

## 3. Results

### 3.1. Clinicopathological Characteristics of Patients

A total of 70 TET patients (30 males and 40 females) aged 29–77 (mean, 56.7 ± 12) years were included in the study. The diameters of the primary tumors were 1.2–11.74 cm. All the cases were classified according to the World Health Organization (WHO) (2015) histological criteria of thymic tumor. The cohort consisted of 11, 13, 9, 7, 10, and 20 cases of type A, type AB, type B1, type B2, type B3, and thymic carcinoma, respectively. All the thymic carcinoma cases were squamous cell carcinomas. According to the Masaoka clinical staging of thymoma, the cases of stages I, II, III, and IV were 32, 4, 20, and 14, respectively. Eleven patients exhibited comorbidity with myasthenia gravis, 37 patients underwent radiotherapy after surgery or biopsy diagnosis, and 21 patients were given chemotherapy after surgery or biopsy diagnosis; cervical lymph node metastasis, classified as type B3, was detected in 1 case of thymoma, and lymph node or other organ metastasis was observed in 13 thymic carcinoma patients.

### 3.2. Expression of PD-L1/PD-1 Protein in TETs

**Figure 1:**
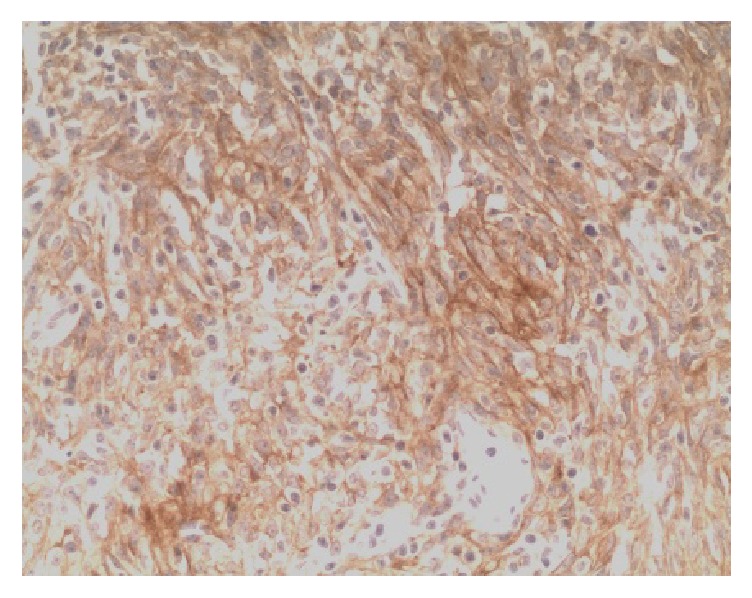
Type A thymoma: PD-L1-positive IHC staining, the tumor cell membrane displayed linear staining from brownish yellow to tan (Envision method, ×200).

**Figure 2:**
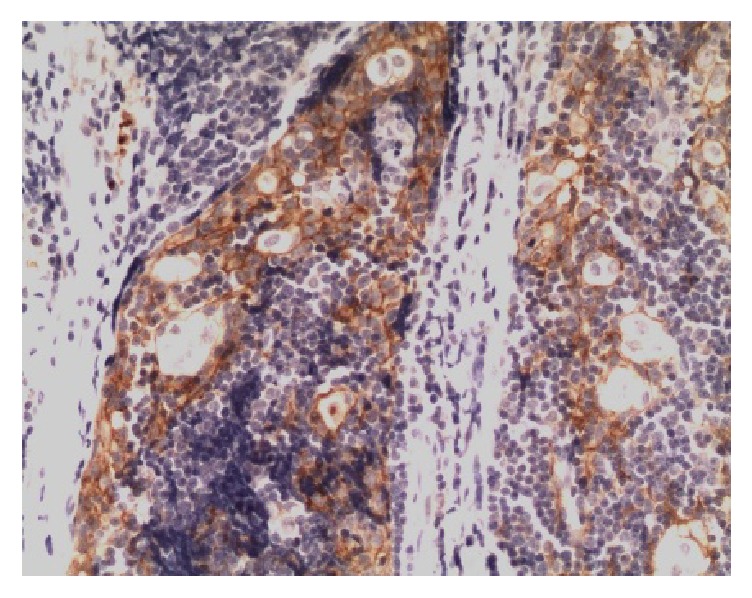
Type B2 thymoma: PD-L1-positive IHC staining, the tumor cell membrane displayed linear staining from brownish yellow to tan (Envision method, ×200).

**Figure 3:**
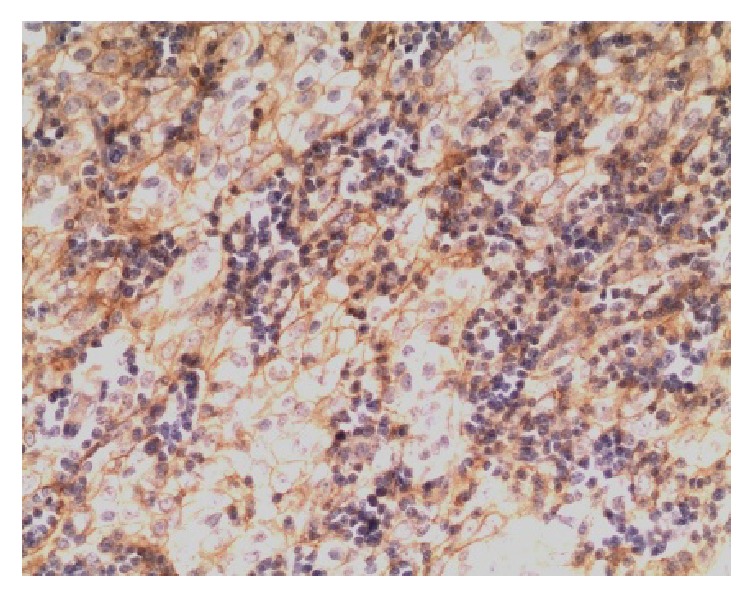
Type B3 thymoma: PD-L1-positive IHC staining, the tumor cell membrane displayed linear staining from brownish yellow to tan (Envision method, ×200).

**Figure 4:**
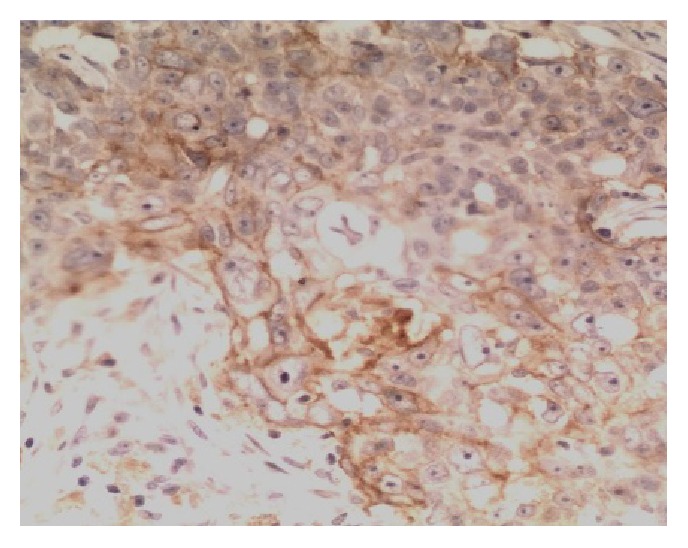
Thymic carcinoma (squamous cell carcinoma): PD-L1-positive IHC staining, the tumor cell membrane displayed linear staining from brownish yellow to tan (Envision method, ×200).

**Figure 5:**
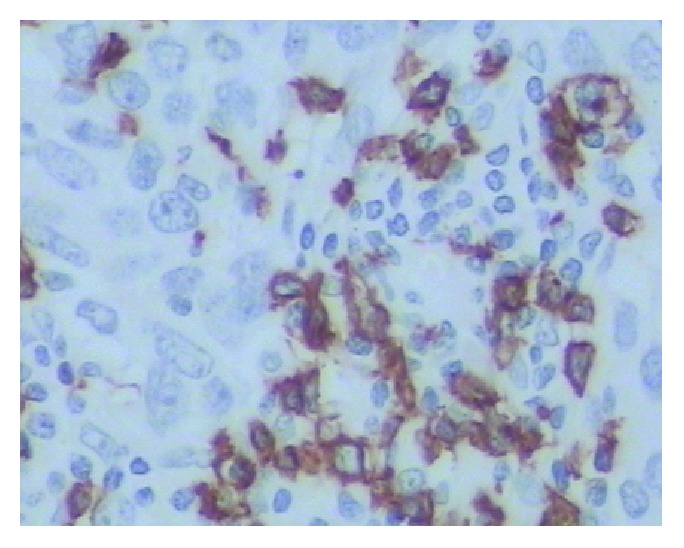
Thymic carcinoma: PD-1-positive IHC staining, the cytoplasmic staining of the interstitial immune cells was positive, and the tumor cells staining was negative (Envision method, ×400).

**Figure 6:**
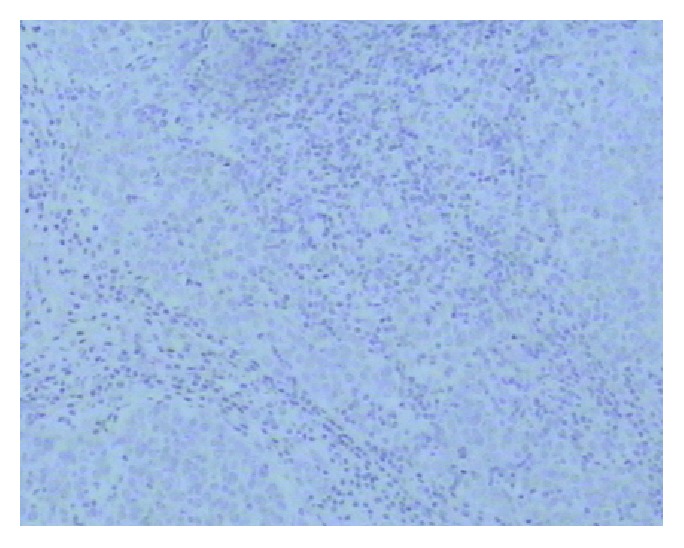
Type C thymoma: PD-1-negative IHC staining (Envision method, ×100).

PD-L1 protein was expressed on the cell membrane of tumor cells that appeared as light yellow, brownish yellow, and tan depending on the expression level (Figures 1–4). PD-1 protein was expressed in the cytoplasm of interstitial lymphocytes (Figures 5 and 6). The positive rate of PD-L1 expression was 54.29% (38/70) in 70 cases of TETs. Among these, the positive rate of PD-L1 protein was 48% (24/50) and 70% (14/20) in thymoma and thymic carcinoma tissues, respectively; however, there was no significant difference in the positive rates between the two groups (*χ*^2^ = 2.786, *P* > 0.05). Considering that the biological behaviors of type B3 thymoma were similar to that of thymic carcinoma, it was classified as thymus carcinoma for analysis. The positive rate of PD-L1 in type B3 thymoma and thymic carcinoma (76.67%, 23/30) was significantly higher than that in other types of thymomas (37.5%, 15/40) (*χ*^2^ = 10.597, *P* < 0.05). In 20 cases of thymic carcinoma tissues, the rate of PD-1 was positive in 13 cases of tumor-infiltrating lymphocytes (TILs) with a positive rate of 65%, and in 14 cases of tumor cells with a positive rate of 70%. In addition, PD-1 was positively correlated with PD-L1 expression in tumor cells (*P* < 0.05, correlation coefficient *r* = 0.663).

### 3.3. Relationship between PD-L1 Protein Expression and Clinicopathological Features in TETs

The PD-L1 protein expression in TETs was not associated with gender, age, tumor size, with/without metastasis, and with/without myasthenia gravis symptoms; however, it was correlated with WHO histological classification, Masaoka-Koga staging, radiotherapy, and chemotherapy. The difference was statistically significant (all *P* < 0.05) (Table 1).

### 3.4. Quantitation of PD-L1 mRNA Expression

Of the 70 cases of TETs, the* PD-L1* mRNA was highly expressed in 17 cases, with positive PD-L1 protein detection and positive coincidence rate of 100%. On the other hand,* PD-L1* mRNA was moderately expressed in 22 cases, of which the PD-L1 protein was positive in 15 cases with a positive coincidence rate of 68.18%. The* PD-L1* mRNA was lowly expressed in 17 cases, in which the PD-L1 protein was positive in only 1 case, with a negative coincidence rate of 94.12%. The concentration and purity of RNA extracted from 14 samples were infinitesimally low, and, hence, these were regarded as unqualified samples and not included in the statistical analysis. Five cases of PD-L1 protein were detected as positive. The coincidence rate of the two detection methods was 85.71%. Spearman correlation analysis showed that* PD-L1* mRNA expression was positively correlated with PD-L1 protein expression (*P* < 0.001, correlation coefficient = 0.745, Table 2).

### 3.5. Analyzing PD-L1 Expression in Different Types of TETs from TCGA Database

**Figure 7:**
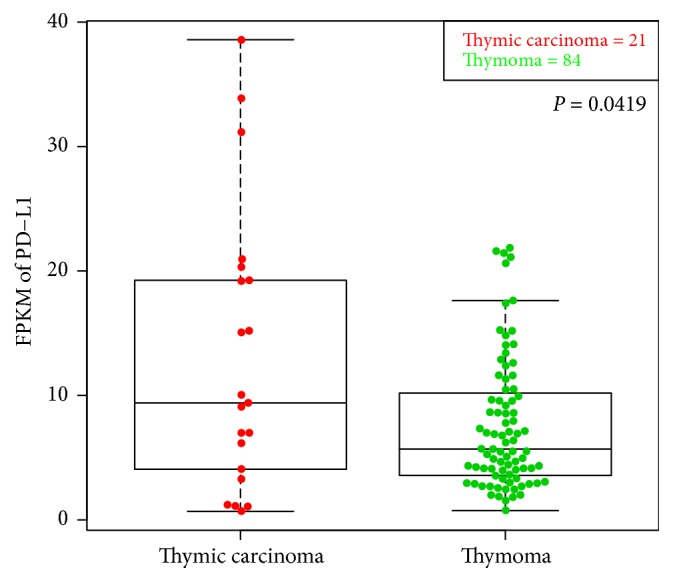
Analyzing PD-L1 expression in different types of TETs from TCGA database. We compared PD-L1 expression in thymoma with that in thymic carcinoma, and the difference was significant (*P* < 0.05).

We obtained 105 TETs cases with detailed clinical information and RNA-seq results from TCGA database. Based on the clinical characteristics, all TET patients (*n* = 105) could be simply divided into two categories, including thymoma (*n* = 84) and thymic carcinoma (*n* = 21). We found that the median PD-L1 expression in patients with thymoma was 5.68, and the median PD-L1 expression in patients with thymic carcinoma was 9.39 (Figure 7). The difference was significant (*P* = 0.0419). The result further suggests that high PD-L1 expression is substantially correlated with malignancy of TET tumors.

## 4. Discussion

The study of the PD-1/PD-L1 pathway in many tumors has been under intensive focus. However, only a few reports about PD-1/PD-L1 in TETs are available in recent years. In this study, we analyzed the expression of PD-L1 protein in 70 cases of TETs. The results showed that the positive expression rate of PD-L1 protein in TETs was 54.29%. Previous studies [10, 11, 13–18] demonstrated that the positive expression rate of PD-L1 protein in thymoma was 18–92%. The results of PD-L1 protein quantitation proposed that the differences may be attributed to the following reasons: (1) the clone number of the PD-L1 antibody varied in different studies. For example, the staining model of PD-L1 antibody (SP142) used in this experiment was from weak to strong staining of the cell membrane, while the PD-L1 antibody with clone numbers 5H1 and 15 provided a diffused and consistent staining model, respectively [10, 13]. Thus, the SP142 staining model of PD-L1 antibody may be optimal for the quantitative assessment, with high reliability. (2) The standards for the interpretation of positive expression were inconsistent. For example, some PD-L1 clone numbers stained not only the tumor cells but also interstitial cells. The two kinds of positive cells were included during interpretation, which led to an increase in the positive rate of TETs [15]. (3) The subjectivity of the scoring was noted in positive interpretation by different observers, and the repeatability was poor. (4) Sample size and sample selection errors, for example, high percentage of type A and type AB thymoma in the cohort, would lead to a low TETs positive rate [13]. (5) The heterogeneity of PD-L1 expression in different sampling sites was associated with the differences.

Moreover, this study, for the first time, used real-time fluorescence qPCR to detect the* PD-L1* mRNA expression in TETs, which also provided a credible basis for the estimation of PD-L1 protein. The results showed that the expression of* PD-L1* mRNA in high- and low-expression groups was in agreement with that by IHC detection; however, the difference primarily occurred in the moderate-expression group, in which the concordance rate with protein was 68.18%. These differential phenomena might be attributed to the following: real-time fluorescence qPCR detected the mRNA expression level in all cells, including tumor and interstitial cells; however, the IHC interpretation of PD-L1 protein was confined to the tumor cells, and the analysis did not include the nontumor lymphocytes that expressed the PD-L1 protein. Furthermore, TETs were different from other solid tumors, especially type B1 and B2 thymoma. The TETs were similar to the normal thymus, with relatively abundant nontumor immune cells. A total of 14 cases of patients with tissues expressing* PD-L1* mRNA did not reveal any unqualified RNA. However, IHC showed positive PD-L1 expression in 5 cases. Although the sensitivity of mRNA detection was higher than that of IHC, it could not clearly identify the same in tumor cells, and the specificity was poor. On the other hand, the RNA was degraded due to the method of sample fixation, the length of time, and the long shelf-life, which led to failed obtaining results. Although a certain degree of subjectivity was noted in the selection of IHC antibody and the judgment of positive intensity and positive threshold, the percentage of false-positive and false-negative results would appear with advantages of simple operation, mature technology, low cost, and improvement in the drawbacks by quality control. Therefore, the IHC method can be used as a detection indicator for PD-L1 in TETs with its unique advantages of simple operation and cost-efficiency.

Studies have found that the positive expression of the PD-L1 protein was an adverse prognostic factor [19, 20]. The high expression of PD-L1 in thymoma can be an independent risk factor for tumor recurrence and predict a poor overall survival [10, 13]. The prognosis of cancer patients was closely related to the clinicopathological features. WHO classification, Masaoka staging, and preoperative treatment remarkably affected the overall survival rate of TETs [11]. However, there are still some paradoxical observations in previous reports [21, 22]. In order to further verify the correlation between the expression of PD-L1 protein and the biological behaviors of TETs, the present study analyzed the relationship between the expression of PD-L1 protein and clinicopathological features of TETs. The results showed that the positive expression of PD-L1 protein was correlated with the WHO histological classification and Masaoka-Koga staging of TETs. The positive expression rate of PD-L1 protein in type B3 thymoma and thymic carcinoma was significantly higher than that in other thymoma subtypes. Furthermore, we reconfirmed our observations by analyzing TCGA data. These results indicated that the expression of PD-L1 protein was correlated with the invasive and malignant degree of the tumors.

A majority of the TETs were composed of a mixture of neoplastic epithelial and nonneoplastic lymphocytes. Moreover, the detection of PD-L1 alone cannot comprehensively determine the prognosis of TETs. PD-1 is primarily expressed on activated T lymphocytes. Studies have shown that an increase of PD-1-positive TILs suggested a poor prognosis [23, 24]. Thymic cancer differed from that of thymoma in both prognosis and molecular phenotype. Based on these above speculations, the relationship between PD-L1 and PD-1 in thymic carcinoma was further evaluated in this study. The results showed that the positive expression rate of TILs PD-1 was 65% in 20 cases of thymic carcinoma. After further analysis of the correlation between PD-1 and PD-L1, we found that the expression of PD-1 and PD-L1 in thymic carcinoma was positively correlated, thereby indicating that TILs PD-1 together with tumor cell PD-L1 can reflect the activation state of the PD-1/PD-L1 pathway. This experiment, for the first time, proved a positive correlation between PD-1 and PD-L1 in thymic carcinoma. Immunotherapy targeting PD-1/PD-L1 exerts antitumor effects in several malignant tumors, and we expected this to be demonstrated in thymic cancers, especially in patients with thymic carcinoma insensitive to chemotherapy. Previous studies showed that PD-L1-positive patients responded better to immune checkpoint inhibitors than the PD-L1-negative patients in other malignant tumors [25, 26]. Based on the above findings and the high expression of PD-L1 protein in thymic carcinoma in the current study, we speculated that the thymic carcinoma with PD-1/PD-L1 could be treated as the target. Nevertheless, this study also had some limitations. The number of samples collected was relatively small due to the rarity of thymic carcinoma. Hence, further prospective studies with large sample size are essential to verify these findings.

The present study also found that PD-L1 protein expression was associated with radiotherapy and chemotherapy; however, the underlying mechanism was yet unknown. Katsuya et al. [17] reported that the expression of PD-L1 in tumor cells and PD-1 in interstitial lymphocytes was increased significantly in TETs undergoing chemotherapy. This result was beneficial for postchemotherapy immunotherapy and proposed the putative combination of chemotherapy and immunotherapy in treating TETs.

## 5. Conclusion

Overall, the current findings indicated that the expressions of PD-L1 protein and mRNA differed in thymoma and thymic carcinomas, and PD-L1 may serve as a potential marker of invasiveness and prognosis. In addition, the high expression of PD-L1 and PD-1 in TETs has also made it possible to clinically adopt the immunotherapy for targeting PD-1/PD-L1.

## Figures and Tables

**Table 1:** Relationship between the expression of PD-L1 protein and clinicopathological features in TETs.

Clinicopathological factors	Case number	PD-L1 expression		Positive rate (%)	*χ* ^2^	*P* value
		+	−			
Gender						
Male	30	20	10	33.33	3.243	0.092
Female	40	18	22	45.00		
Age						
≤50 years	16	7	9	43.75	0.928	0.399
>50 years	54	31	23	57.41		
Tumor size						
≤4 cm	41	22	19	53.66	0.016	1.000
>4 cm	29	16	13	55.17		
Histological classification						
Type A	11	4	7	36.36	20.648	0.001^*∗*^
Type AB	13	2	11	15.39		
Type B1	9	3	6	33.33		
Type B2	7	6	1	85.71		
Type B3	10	9	1	90.00		
Type C thymic carcinoma	20	14	6	70.00		
Masaoka-Koga staging						
Stage I	32	10	22	31.25	12.402	0.004^*∗*^
Stage II	4	3	1	75.00		
Stage III	20	15	5	75.00		
Stage IV	14	10	4	71.42		
With/without myasthenia gravis						
Yes	11	7	4	63.64	0.460	0.533
No	59	31	28	52.54		
Metastasis						
Yes	14	10	4	71.43	2.072	0.231
No	56	28	28	50.00		
Radiotherapy						
Yes	37	26	11	70.27	8.081	0.008^*∗*^
No	33	12	21	36.36		
Chemotherapy						
Yes	21	16	5	76.19	5.800	0.02^*∗*^
No	49	22	27	44.90		

^*∗*^ *P* < 0.05.

**Table 2:** Correlation between mRNA and protein expression of PD-L1.

*PD-L1* mRNA expression	PD-L1 protein expression		Coincidence rate (%)	*χ* ^2^	*P* value
	+	−			
High expression	17	0	100		
Moderate expression	15	7	68.18	32.392	<0.001
Low expression	1	16	94.12		

≤Spearman correlation coefficient *r* = 0.745.

## References

[1] Detterbeck F., Parsons A. M., Patterson G. A., Cooper J. D., Deslauriers J. (2008). Thymic tumor: a review of current diagnosis, classification, and treatment. *Thoracic and Esophageal Surgery*.

[2] Detterbeck F. C., Parsons A. M. (2011). Management of stage I and II thymoma. *Thoracic Surgery Clinics*.

[3] Han L., Li R., Chen X. (2014). The expression and clinical significance of B7 family molecules and their receptor PD-1 in human NK/T-cell lymphoma. *Chinese Journal of Clinical Oncology*.

[4] Lages C. S., Lewkowich I., Sproles A., Wills-Karp M., Chougnet C. (2010). Partial restoration of T-cell function in aged mice by in vitro blockade of the PD-1/PD-L1 pathway. *Aging Cell*.

[5] Thompson R. H., Kuntz S. M., Leibovich B. C. (2006). Tumor B7-H1 is associated with poor prognosis in renal cell carcinoma patients with long-term follow-up. *Cancer Research*.

[6] Dong H., Strome S. E., Salomao D. R. (2002). Tumor-associated B7-H1 promotes T-cell apoptosis: a potential mechanism of immune evasion. *Nature Medicine*.

[7] Chen Y.-B., Mu C.-Y., Huang J.-A. (2012). Clinical significance of programmed death-1 ligand-1 expression in patients with non-small cell lung cancer: A 5-year-follow-up study. *Tumori*.

[8] Velcheti V., Schalper K. A., Carvajal D. E. (2014). Programmed death ligand-1 expression in non-small cell lung cancer. *Laboratory Investigation*.

[9] Brown J. A., Dorfman D. M., Ma F.-R. (2003). Blockade of programmed death-1 ligands on dendritic cells enhances T cell activation and cytokine production. *The Journal of Immunology*.

[10] Padda S. K., Riess J. W., Schwartz E. J. (2015). Diffuse high intensity PD-L1 staining in thymic epithelial tumors. *Journal of Thoracic Oncology*.

[11] Katsuya Y., Fujita Y., Horinouchi H., Ohe Y., Watanabe S., Tsuta K. (2015). Immunohistochemical status of PD-L1 in thymoma and thymic carcinoma. *Lung Cancer*.

[12] Taube J. M., Klein A., Brahmer J. R. (2014). Association of PD-1, PD-1 ligands, and other features of the tumor immune microenvironment with response to anti-PD-1 therapy. *Clinical Cancer Research*.

[13] Yokoyama S., Miyoshi H., Nishi T. (2016). Clinicopathologic and Prognostic Implications of Programmed Death Ligand 1 Expression in Thymoma Presented at the Sixteenth World Conference on Lung Cancer, Denver, CO, September 6-9, 2015.. *The Annals of Thoracic Surgery*.

[14] Tiseo M., Damato A., Longo L. (2017). Analysis of a panel of druggable gene mutations and of ALK and PD-L1 expression in a series of thymic epithelial tumors (TETs). *Lung Cancer*.

[15] Marchevsky A. M., Walts A. E. (2017). PD-L1, PD-1, CD4, and CD8 expression in neoplastic and nonneoplastic thymus. *Human Pathology*.

[16] Yokoyama S., Miyoshi H., Nakashima K. (2016). Prognostic value of programmed death ligand 1 and programmed death 1 expression in thymic carcinoma. *Clinical Cancer Research*.

[17] Katsuya Y., Horinouchi H., Asao T. (2016). Expression of programmed death 1 (PD-1) and its ligand (PD-L1) in thymic epithelial tumors: Impact on treatment efficacy and alteration in expression after chemotherapy. *Lung Cancer*.

[18] Weissferdt A., Fujimoto J., Kalhor N. (2017). Expression of PD-1 and PD-L1 in thymic epithelial neoplasms. *Modern Pathology*.

[19] Wu C., Zhu Y., Jiang J., Zhao J., Zhang X.-G., Xu N. (2006). Immunohistochemical localization of programmed death-1 ligand-1 (PD-L1) in gastric carcinoma and its clinical significance. *Acta Histochemica*.

[20] Hamanishi J., Mandai M., Iwasaki M. (2007). Programmed cell death 1 ligand 1 and tumor-infiltrating CD8^+^ T lymphocytes are prognostic factors of human ovarian cancer. *Proceedings of the National Acadamy of Sciences of the United States of America*.

[21] Padda S. K., Riess J. W., Schwartz E. J. (2015). Diffuse High Intensity PD–L1 Staining in Thymic Epithelial Tumors. *Journal of Thoracic Oncology*.

[22] Arbour K. C. (2017). Expression of PD-L1 and other immunotherapeutic targets in thymic epithelial tumors. *PLoS One*.

[23] Muenst S., Soysal S. D., Gao F., Obermann E. C., Oertli D., Gillanders W. E. (2013). The presence of programmed death 1 (PD-1)-positive tumor-infiltrating lymphocytes is associated with poor prognosis in human breast cancer. *Breast Cancer Research and Treatment*.

[24] Thompson R. H., Dong H., Lohse C. M. (2007). PD-1 is expressed by tumor-infiltrating immune cells and is associated with poor outcome for patients with renal cell carcinoma. *Clinical Cancer Research*.

[25] Philips G. K., Atkins M. (2015). Therapeutic uses of anti-PD-1 and anti-PD-L1 antibodies. *International Immunology*.

[26] Meng X., Huang Z., Teng F., Xing L., Yu J. (2015). Predictive biomarkers in PD-1/PD-L1 checkpoint blockade immunotherapy. *Cancer Treatment Reviews*.

